# Supplementary material for: The Analysis of the Mycobiota in Plastic Polluted Soil Reveals a Reduction in Metabolic Ability
Source: J Fungi (Basel). 2022 Nov 25;8(12):1247. doi: 10.3390/jof8121247 (PMC9785340; doi:10.3390/jof8121247)
Supplement: Supplementary file 1 [file jof-08-01247-s001.zip › Figure S1.pdf]

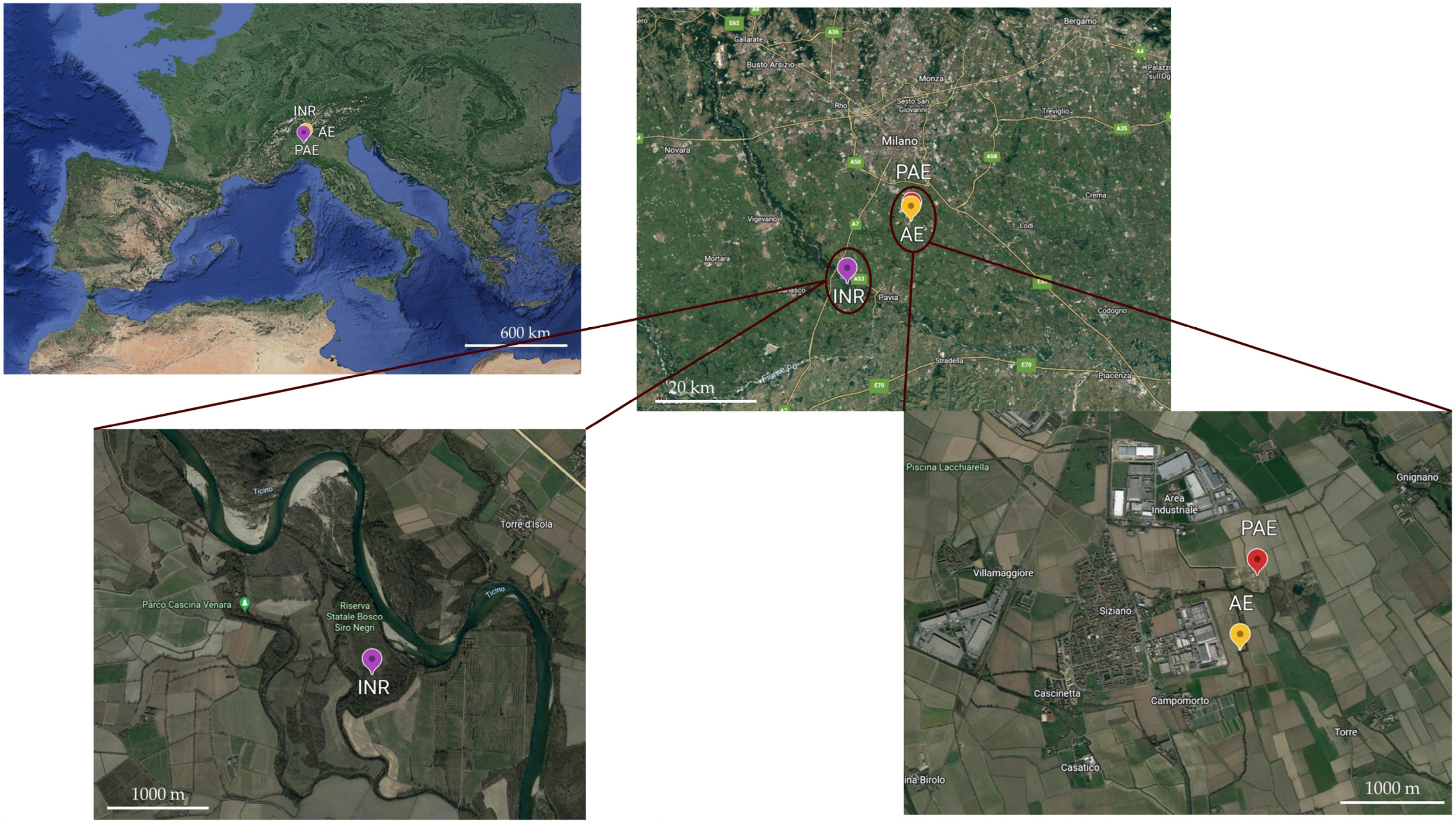

Figure S1. Map of sampled sites. PAE: Polluted AgroEcosystem; AE: AgroEcosystem; INR: Integral Natural Reserve.
