# Supplementary material for: The Analysis of the Mycobiota in Plastic Polluted Soil Reveals a Reduction in Metabolic Ability
Source: J Fungi (Basel). 2022 Nov 25;8(12):1247. doi: 10.3390/jof8121247 (PMC9785340; doi:10.3390/jof8121247)
Supplement: Supplementary file 1 [file jof-08-01247-s001.zip › Table S1.pdf]

Table S1. Carbon substrates of Biolog EcoPlate™ divided into six categories

Amines	Amino acids	Carbohydrates	Carboxylic acids	Phenolic compounds	Polymers
Phenylethylamine	L-Arginine	β-Methyl-D-Glucoside	D-Galactonic Acid γ-Lactone	Pyruvic Acid Methyl Ester	Tween 40
Putrescine	L-Asparagine	D-Xylose	D-Galacturonic Acid	D-Mannitol	Tween 80
	Lphenylalanine	i-Erythritol	2-Hydroxy Benzoic Acid	N-Acetyl-D-Glucosamine	α -Cyclodextrin
	L-Serine	D,L-α- Glycerol Phosphate	4-Hydroxy Benzoic Acid		Glycogen
	L-Threonine	D-Cellobiose	γ-Amino Butyric Acid		
	Glycyl-Lglutamic Acid	Glucose-1-Phosphate	D-Glucosaminic Acid		
		α-D-Lactose	Itaconic Acid		
			α-Keto Butyric Acid		
			D-Malic Acid		
